# Body Mass Index and Mortality in Korean Intensive Care Units: A Prospective Multicenter Cohort Study

**DOI:** 10.1371/journal.pone.0090039

**Published:** 2014-04-18

**Authors:** So Yeon Lim, Won-Il Choi, Kyeongman Jeon, Eliseo Guallar, Younsuck Koh, Chae-Man Lim, Shin Ok Koh, Sungwon Na, Young-Joo Lee, Seok Chan Kim, Ick Hee Kim, Je Hyeong Kim, Jae Yeol Kim, Jaemin Lim, Chin Kook Rhee, Sunghoon Park, Ho Cheol Kim, Jin Hwa Lee, Jisook Park, Gee Young Suh, Validation of Simplified acute physiology score 3 in Korean Intensive care unit (VSKI) study group, the Korean study group on respiratory failure (KOSREF)

**Affiliations:** 1 Division of Pulmonary and Critical Care Medicine, Department of Medicine, Samsung Medical Center, Sungkyunkwan University School of Medicine, Seoul, Korea; 2 Division of Pulmonary and Critical Care Medicine, Department of Medicine, Keimyung University, Dongsan Hospital, Daegu, Korea; 3 Department of Critical Care Medicine, Samsung Medical Center, Sungkyunkwan University School of Medicine, Seoul, Korea; 4 Department of Epidemiology and Welch Center for Prevention, Epidemiology, and Clinical Research, Johns Hopkins University Bloomberg School of Public Health, Baltimore, Maryland, United States of America; 5 Division of Pulmonary and Critical Care Medicine, Department of Medicine, Asan Medical Center, University of Ulsan College of Medicine, Seoul, Korea; 6 Department of Anesthesiology and Pain Medicine, and Anesthesia and Pain Research Institute, Yonsei University College of Medicine, Seoul, Korea; 7 Department of anesthesiology, Aju university college of medicine, Suwon, Korea; 8 Division of Pulmonary and Critical Care Medicine, Department of Medicine, Seoul St. Mary's Hospital, Catholic University of Korea, Seoul, Korea; 9 Department of Surgery, Chungju hospital, School of medicine of Konkuk university, Chungju, Korea; 10 Division of Pulmonary, Sleep, and Critical Care Medicine, Department of Medicine, Korea University Ansan Hospital, Ansan, Korea; 11 Division of Pulmonary and Critical Care Medicine, Department of Medicine, Chung-Ang University College of Medicine, Seoul, Korea; 12 Division of Pulmonary and Critical Care Medicine, Department of Medicine, Gangneung Asan Hospital, University of Ulsan Medical College of medicine, Gangneung, Korea; 13 Department of Pulmonary, Allergy, and Critical Care Medicine, Hallym University Sacred Heart Hospital, Anyang, Korea; 14 Division of Pulmonary and Critical Care Medicine, Department of Medicine, College of Medicine, Gyeongsang Institute of Health Sciences, Gyeongsang National University, Jinju, Korea; 15 Division of Pulmonary and Critical Care Medicine, Department of Medicine, Ewha Womans University School of Medicine, Seoul, Korea; 16 Department of Multimedia, Seoul Women's University, Seoul, Korea; Tulane School of Public Health and Tropical Medicine, United States of America

## Abstract

**Background:**

The level of body mass index (BMI) that is associated with the lowest mortality in critically ill patients in Asian populations is uncertain. We aimed to examine the association of BMI with hospital mortality in critically ill patients in Korea.

**Methods:**

We conducted a prospective multicenter cohort study of 3,655 critically ill patients in 22 intensive care units (ICUs) in Korea. BMI was categorized into five groups: <18.5, 18.5 to 22.9, 23.0 to 24.9 (the reference category), 25.0 to 29.9, and ≥30.0 kg/m^2^.

**Results:**

The median BMI was 22.6 (IQR 20.3 to 25.1). The percentages of patients with BMI<18.5, 18.5 to 22.9, 23.0 to 24.9, 25.0 to 29.9, and ≥30.0 were 12, 42.3, 19.9, 22.4, and 3.3%, respectively. The Cox-proportional hazard ratios with exact partial likelihood to handle tied failures for hospital mortality comparing the BMI categories <18.5, 18.5 to 22.9, 25.0 to 29.9, and ≥30.0 with the reference category were 1.13 (0.88 to 1.44), 1.03 (0.84 to 1.26), 0.96 (0.76 to 1.22), and 0.68 (0.43 to 1.08), respectively, with a highly significant test for trend (*p* = 0.02).

**Conclusions:**

A graded inverse association between BMI and hospital mortality with a strong significant trend was found in critically ill patients in Korea.

## Introduction

Body mass index (BMI) is the most commonly used surrogate marker of overall adiposity. In the general population, both low and high BMI are associated with increased mortality [1], [2], [3], [4], [5]. In critically ill patients, however, being underweight is an established prognostic factor of mortality but the impact of being overweight or obese is still controversial [6], [7], [8], [9], [10], [11].

Few large prospective studies have evaluated the association of BMI with mortality in medical and surgical intensive care units (ICUs) using consistent cutoff values [12]. Furthermore, most studies of BMI and mortality in critically ill patients have been conducted in Western populations [10], [12], [13]. Because Asians have a different body build, a different fat distribution, and a higher percentage of body fat for the same BMI compared with Caucasians [14], [15], previous studies in Western populations may not apply to Asians. Therefore, the aim of this prospective study was to investigate the association between BMI and hospital mortality in a large sample of 3,655 critically ill patients in 22 intensive care units (ICUs) in Korea.

## Materials and Methods

The study was approved by each hospital's institutional review board (Samsung Medical Center, Dongsan Hospital, Asan Medical Center, Yonsei University College of Medicine, Aju university college of medicine, Seoul St. Mary's Hospital, Chungju hospital, Korea University Ansan Hospital, Chung-Ang University College of Medicine, Gangneung Asan Hospital, Hallym University Sacred Heart Hospital, Gyeongsang National University, Ewha Womans University School of Medicine, The Armed Forces Capital Hospital, and Bundang CHA hospital). The requirement for informed consent was waived because the study was based on routinely collected clinical data.

We used data from the “Validation of simplified acute physiology score 3 in Korean ICUs” (VSKI) study cohort [16]. VSKI was a prospective multicenter cohort study for the validation of the simplified acute physiology score 3 (SAPS 3) in Korean ICU patients performed by the Korean Study group on Respiratory Failure (KOSREF) between July 1^st^, 2010 and January 31^th^, 2011 [16]. The VSKI included 22 ICUs (medical = 14, surgical = 6 and multidisciplinary = 2) in tertiary or university-affiliated hospitals [16].

### Patients

The patient population included all of the patients admitted to the participating ICUs during the study period. We excluded patients if they were less than 17 years old or if hospital mortality was uncertain. The patients transferred from other participating ICUs were also excluded. For patients with two or more admissions to the ICU during the same hospital stay, we only used data from the first admission.

### Data collection

The patients' data were recorded prospectively in a web-based database. We obtained data on demographic characteristics (age, sex, body weight and height), underlying disease, SAPS 3, sequential organ failure assessment (SOFA) score, severe sepsis or septic shock at ICU admission, acute respiratory distress syndrome (ARDS) at ICU admission, admission category (medical or surgical), admission diagnosis, organ support (mechanical ventilation, continuous renal replacement therapy (CRRT), and use of vasopressors), length of ICU stay and hospital stay, and mortality at ICU and hospital discharge. The weight and height were measured upon ICU admission. The BMI (weight in kilograms divided by height in meters squared) values were calculated and categorized into 5 groups: <18.5, 18.5 to 22.9, 23.0 to 24.9 (the reference category), 25.0 to 29.9, and ≥30.0 kg/m^2^.

### Statistical analysis

The statistical analyses were performed using STATA 12.0 (StataCorp LP, Texas, USA). The data were summarized using medians and interquartile ranges (IQRs) for continuous variables and frequencies and percentages for categorical variables. The primary analysis was based on Cox proportional hazard models with exact partial likelihood to handle tied failures (*exactp* option in Stata) [17]. We estimated the hazard ratios and 95% confidence intervals for hospital mortality comparing BMI categories with the reference category (23.0 to 24.9). We calculated a crude model and a model adjusted for age, sex, and all variables that were associated with mortality with *p*<0.25 in univariate analyses. Tests for trend were calculated by introducing in the hazard models a variable with the median BMI in each category. As a sensitivity analysis, we repeated the analysis using a discrete-time hazard model [18], with similar results (not shown). Finally, *p* values<0.05 were considered to be statistically significant.

## Results

A total of 3,655 patients were included in the analysis. The median age was 62 years (IQR 49 to 72), and 63.8% (2,332/3,655) of the study participants were men. The percentage of medical patients was 59% (2,156/3,655), and the most common reason for ICU admission was observational intensive care (42.6%, 1,558/3,655), followed by respiratory problems (18.3%, 669/3,655). The median BMI was 22.6 (IQR 20.3 to 25.1). Table 1 shows the baseline characteristics of the study patients by BMI category. The percentages of patients with BMI<18.5, 18.5 to 22.9, 23.0 to 24.9, 25.0 to 29.9, and ≥30.0 were 12, 42.3, 19.9, 22.4, and 3.3%, respectively.

**Table 1:** Baseline characteristics according to body mass index.

	<18.5	18.5–22.9	23.0–24.9	25.0–29.9	≥30.0	Total
**Number**	440 (12)	1547 (42.3)	727 (19.9)	820 (22.4)	121 (3.3)	3655
**Age**	67 (51–76)	61 (49–72)	62 (50–71)	61 (50–70)	57 (44–69)	62 (49–72)
**Male**	263 (59.8)	1004 (64.9)	485 (66.7)	520 (63.4)	60 (49.6)	2332 (63.8)
**Comorbidities**						
Cirrhosis	32 (7.3)	143 (9.2)	67 (9.2)	101 (12.3)	19 (15.7)	362 (9.9)
Cardiovascular disease	161 (36.6)	574 (37.1)	312 (42.9)	393 (47.9)	57 (47.1)	1497 (41)
CPF*	34 (7.7)	29 (1.9)	13 (1.8)	11 (1.3)	1 (0.8)	88 (2.4)
DM	85 (19.3)	332 (21.5)	149 (20.5)	196 (23.9)	35 (28.9)	797 (21.8)
CRF	45 (10.2)	139 (9)	56 (7.7)	57 (7)	9 (7.4)	306 (8.4)
Cancer	134 (30.5)	562 (36.3)	271 (37.3)	317 (38.7)	43 (35.5)	1327 (36.3)
**Status at ICU admission**						
SS or septic shock	98 (22.3)	256 (16.6)	111 (15.3)	119 (14.5)	26 (21.5)	610 (16.7)
ARDS	46 (10.5)	112 (7.4)	55 (7.6)	38 (4.6)	6 (5)	257 (7)
**Admission category**						
Medical	321 (73)	941 (60.8)	386 (53.1)	439 (53.5)	69 (57)	2156 (59)
Surgical	119 (27.1)	606 (39.2)	341 (46.9)	381 (46.5)	52 (43)	1499 (41)
**Reason for ICU admission**						
Observational†	143 (32.5)	672 (43.4)	326 (44.8)	374 (45.6)	43 (35.5)	1558 (42.6)
Cardiovascular	72 (16.4)	190 (12.3)	90 (12.4)	108 (13.2)	22 (18.2)	482 (13.2)
Digestive	20 (4.6)	88 (5.6)	31 (4.3)	45 (5.5)	7 (5.8)	189 (5.2)
Hepatic failure	8 (1.8)	53 (3.4)	33 (4.5)	49 (6)	12 (9.9)	155 (4.2)
Neurologic	17 (3.9)	93 (6)	42 (5.8)	51 (6.2)	3 (2.5)	206 (5.6)
Renal	7 (1.6)	32 (2.1)	14 (1.9)	11 (1.3)	4 (3.3)	68 (1.9)
Respiratory	147 (33.4)	276 (17.8)	125 (17.2)	104 (12.7)	17 (14.1)	669 (18.3)

Continuous variables are presented as medians (interquartile ranges).

Categorical variables are presented as frequencies (%).

*CPF included the condition of permanent shortness of breath on light activity due to pulmonary disease, and functionally, the patients are unable to work, to climb stairs or perform household duties.

† Observational reason for ICU admission was defined as the preparation for routine post-surgery care including simple weaning from ventilator after surgery.

CPF, chronic pulmonary disease; DM, diabetes mellitus; CRF, chronic renal failure; ICU, intensive care unit; SS, severe sepsis; ARDS, acute respiratory distress syndrome.

The severity and outcomes according to BMI were described in Table 2. The median SAPS 3 score was 54 (IQR 43 to 66), the predicted mortality was 23.9% (IQR 8.9 to 48.3), and the median SOFA score was 6 (IQR 3 to 10). The ICU and hospital mortality were 15.1% (551/3,655) and 21.5% (786/3,655), respectively. The highest ICU and hospital mortalities were observed in the BMI<18.5 category (20.2 and 30.2%, respectively).

**Table 2:** Severity and outcomes according to body mass index.

	<18.5	18.5–22.9	23.0–24.9	25.0–29.9	≥30.0	Total
**SAPS 3**	58 (48–71)	54 (43–66)	52 (41–63)	52 (42–65)	54 (43–69)	54 (43–66)
**PDR**	31.5 (14.5–58.5)	23.9 (8.9–48.3)	20.5 (7.2–41.9)	20.5 (8–46.2)	23.9 (8.9–54.5)	23.9 (8.9–48.3)
**SOFA**	7 (4–11)	6 (3–10)	6 (3–10)	6 (3–10)	7 (3–12)	6 (3–10)
**ICU LOS**	5 (3–11)	4 (2–9)	4 (2–7)	4 (2–8)	4 (2–9)	4 (2–8)
**Hospital LOS**	20 (10–38)	19 (11–36)	18 (10–32)	17 (10–32)	19 (10–38)	18 (11–34)
**MV**	226 (51.4)	579 (37.4)	276 (38)	336 (41)	49 (40.5)	1466 (40.1)
**CRRT**	37 (8.4)	141 (9.1)	52 (7.2)	69 (8.4)	20 (16.5)	319 (8.7)
**Vasopressor**	179 (40.7)	450 (29.1)	196 (27)	240 (29.3)	37 (30.6)	1102 (30.2)
**ICU mortality**	89 (20.2)	243 (15.7)	93 (12.8)	109 (13.3)	17 (14.1)	551 (15.1)
**Hospital mortality**	133 (30.2)	345 (22.3)	132 (18.2)	154 (18.8)	23 (18.2)	786 (21.5)

Continuous variables are presented as medians (interquartile ranges).

Categorical variables are presented as frequencies (%).

SAPS, simplified acute physiology score; PDR, predicted death rate; SOFA, sequential organ failure assessment; ICU, intensive care unit; LOS, length of stay; MV, mechanical ventilation; CRRT, continuous renal replacement therapy.

**Figure 1:**
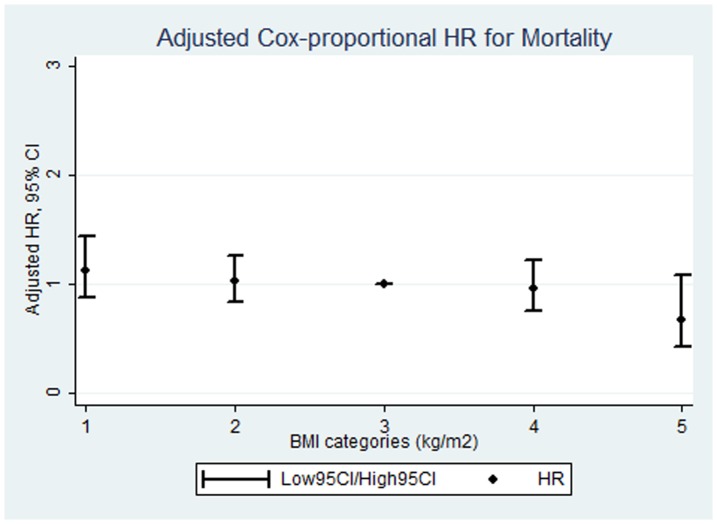
Multivariable-adjusted Cox-proportional hazard ratios with exact partial likelihood for hospital mortality comparing body mass index categories. Multivariable adjusted Cox-proportional hazard ratios with exact partial likelihood and 95% confidence intervals are shown in this figure, illustrating a graded inverse association between BMI and hospital mortality with a strong significant trend (*p* = 0.02).

The Cox-proportional hazard ratios with exact partial likelihood for hospital mortality comparing the BMI categories to the reference category were shown in Table 3. Compared with patients with BMI 23.0 to 24.9, the unadjusted hazard ratios (95% confidence intervals) for hospital mortality in the participants with BMI<18.5, 18.5 to 22.9, 25.0 to 29.9, and ≥30.0 were 1.43 (1.12 to 1.82), 1.15 (0.94 to 1.4), 1.08 (0.85 to 1.36), and 0.83 (0.52 to 1.3), respectively. After adjusting for age, sex, SOFA, chronic pulmonary failure, diabetes mellitus, cancer, severe sepsis or septic shock at ICU admission, ARDS at ICU admission, admission category, use of CRRT, mechanical ventilation, and use of vasopressors, there was a graded inverse association between BMI and hospital mortality. The hazard ratios for the BMI categories <18.5, 18.5 to 22.9, 25.0 to 29.9, and ≥30.0 were 1.13 (0.88 to 1.44), 1.03 (0.84 to 1.26), 0.96 (0.76 to 1.22), and 0.68 (0.43 to 1.08), respectively (Fig. 1), with a highly significant test for trend (*p* = 0.02).

**Table 3:** Cox-proportional hazard ratios with exact partial likelihood and 95% confidence intervals for hospital mortality according to body mass index categories.

	<18.5	18.5–22.9	23.0–24.9	25.0–29.9	≥30.0
**Total (N = 3655)**					
Number of deaths (%)	133 (30.2)	345 (22.3)	132 (18.2)	154 (18.8)	23 (18.2)
Unadjusted HR	1.43	1.15	1	1.08	0.83
(95% CI)	(1.12–1.82)	(0.94–1.4)	(reference)	(0.85–1.36)	(0.52–1.3)
Adjusted HR*	1.13	1.03	1	0.96	0.68
(95% CI)	(0.88–1.44)	(0.84–1.26)	(reference)	(0.76–1.22)	(0.43–1.08)
**Patients with cardiovascular disease (N = 1497)**					
Number of deaths (%)	161 (10.8)	574 (38.3)	312 (20.8)	393 (26.3)	57 (3.8)
Unadjusted HR	1.24	0.94	1	0.98	0.68
(95% CI)	(0.86–1.8)	(0.7–1.26)	(reference)	(0.7–1.37)	(0.34–1.38)
Adjusted HR†	1.07	0.91	1	0.98	0.51
(95% CI)	(0.73–1.56)	(0.67–1.23)	(reference)	(0.7–1.38)	(0.25–1.05)
**Patients with acute respiratory failure (N = 669)**					
Number of deaths (%)	147 (22)	276 (41.3)	125 (18.7)	104 (15.6)	17 (2.5)
Unadjusted HR	0.94	1.05	1	0.92	0.82
(95% CI)	(0.64–1.37)	(0.75–1.46)	(reference)	(0.6–1.42)	(0.33–2.07)
Adjusted HR§	0.94	0.99	1	0.75	0.68
(95% CI)	(0.64–1.38)	(0.7–1.38)	(reference)	(0.48–1.16)	(0.26–1.73)
**Surgical patients (N = 1499)**					
Number of deaths (%)	119 (7.9)	606 (40.4)	341 (22.8)	381 (25.4)	52 (3.5)
Unadjusted HR	1.76	1.33	1	1.49	1.36
(95% CI)	(0.86–3.6)	(0.76–2.32)	(reference)	(0.81–2.76)	(0.52–3.52)
Adjusted HR‡	2.09	1.29	1	1.38	1.3
(95% CI)	(1.01–4.36)	(0.74–2.27)	(reference)	(0.74–2.58)	(0.49–3.42)
**Medical patients (N = 2156)**					
Number of deaths (%)	321 (14.9)	941 (43.7)	386 (17.9)	439 (20.4)	69 (3.2)
Unadjusted HR	1.1	1.01	1	1.02	0.82
(95% CI)	(0.85–1.43)	(0.81–1.26)	(reference)	(0.79–1.31)	(0.49–1.39)
Adjusted HR#	1.04	0.98	1	0.9	0.55
(95% CI)	(0.8–1.36)	(0.78–1.21)	(reference)	(0.69–1.16)	(0.32–0.94)

Categorical variables are presented as frequencies (%).

HR, hazard ratio; CI, confidence interval.

*Adjusted for age, sex, SOFA, CPF, DM, cancer, severe sepsis or septic shock at ICU admission, ARDS at ICU admission, admission category, use of CRRT, mechanical ventilation, and use of vasopressors.

† Adjusted age, sex, SOFA, DM, cancer, severe sepsis or septic shock at ICU admission, ARDS at ICU admission, admission category, use of CRRT, mechanical ventilation, and use of vasopressors.

§ Adjusted age, sex, SOFA, cirrhosis, CPF, cancer, severe sepsis or septic shock at ICU admission, ARDS at ICU admission, admission category, use of CRRT, mechanical ventilation, and use of vasopressors.

‡ Adjusted age, sex, SOFA, cirrhosis, cancer, severe sepsis or septic shock at ICU admission, ARDS at ICU admission, use of CRRT, mechanical ventilation, and use of vasopressors.

# Adjusted age, sex, SOFA, cirrhosis, CPF, cancer, severe sepsis or septic shock at ICU admission, ARDS at ICU admission, use of CRRT, mechanical ventilation, and use of vasopressors.

This trend was consistently observed, even after categorizing BMI into nine equally spaced groups (<17.0, 17.0 to 18.9, 19.0 to 20.9, 21 to 22.9, 23 to 24.9, 25 to 26.9, 27 to 28.9, 29 to 30.9, and ≥31) (*p*<0.001) (see Figure S1, Table S1, Table S2 and Table S3).

## Discussion

In this large, prospective cohort study, a graded inverse relationship between BMI and hospital mortality with a highly significant test for trend was found in a large cohort of critically ill patients in Korea. In multivariable-adjusted analyses, the hazard ratio for hospital mortality comparing the highest with the lowest category of BMI was 1.7 with Cox-proportional hazard model, with a highly significant trend across categories. Obesity paradox was observed in the patients with cardiovascular disease and medical patients

Most studies of the association between BMI and mortality after critical care have been conducted in Western populations [6], [8], [10], [12], [13], [19], [20], [21], [22], [23], [24], [25], [26]. Although there is a degree of controversy, previous studies in ICU patients have shown that low BMI was independently associated with higher mortality [19], [21], [24], and recently, a meta-analysis concluded that low BMI was associated with increased hospital mortality [10]. However, Asians tend to have a more slender body build than Westerns, and slender subjects tend to have less muscle mass [27], [28]. In addition, Asian populations may have a different fat distribution pattern than Western populations and may be more prone to central obesity, even at low BMI levels [15]. Such considerations raise the possibility that the risks associated with low levels of BMI in ICU patients are greater in Asian populations [15]. The only study of BMI and mortality in ICU patients was also conducted in Korea [11]. This study concluded that BMI did not have a significant influence on ICU mortality, but the study was limited by its retrospective design, the inclusion of only medical patients staying >72 hours from admission to the ICU, and the lack of data on hospital mortality. Our study extends the observation of an inverse association between BMI and hospital mortality to a large sample of Asian patients and indicates that BMI is an inverse predictor of short-term death in critically ill patients.

In this study, we categorized BMI into 5 groups (<18.5, 18.5 to 22.9, 23.0 to 24.9, 25.0 to 29.9 and ≥30), as recommended by the collaboration with WHO Western Pacific Regional Office to establish a more appropriate classification of BMI for Asians [14]. Although country-specific cut-off values have been developed in several Asian countries [14], [29], no specific cut-offs have been developed in Korea. These results, however, were consistent when we used alternative categorizations of BMI. In a large study of BMI and mortality in the general population in Korea, Jee et al [30] reported that the risk of death from any cause was lowest among subjects with a BMI of 23.0 to 24.9. As a consequence, we established the group with BMI 23.0 to 23.9 as the reference category in our study. The different mortality patterns with respect to BMI in the general Korean population (J-shaped) and in our study of critically ill patients (inverse association) suggest that the mechanisms relating BMI and mortality are different in long-term outpatient studies and in short-term studies of critically ill patients.

In a large prospective multicenter cohort study of patients with coronary artery disease, patients with normal weight but with central obesity had worse long-term survival than patients with other adiposity patterns [31]. In this study, being overweight or obese by BMI criteria was not associated with higher mortality in the absence of central obesity. These findings support the concept that in patients with chronic conditions BMI may not be a reliable marker of adiposity and clinicians should consider this limitation when assessing the impact of BMI on mortality.

Although it has been suggested that the excess risk of mortality at low BMI in critically ill patients is an artifact caused by the inadequate control of preexisting diseases, the increased odds ratio associated with low BMI remained elevated and highly statistically significant and even in the medical patients there was no increased odds ratio associated with low BMI, reducing the risk of reverse causation. The increased risk of mortality at low BMI levels is likely due to low protein and energy intake, malnutrition, cachexia, and inadequate nutritional reserve resulting in the inability to compensate for the stress of critical illness [24].

In a general population of 1,213,829 Koreans, Jee et al [30] reported that lower BMI compared with the BMI category of 23.0 to 24.9 was linked to the risk of death from respiratory causes. Another large study of 1.1 million persons recruited in Asia also reported that there was a strong association observed between low BMI compared with BMI 22.6 to 27.5 and death from respiratory diseases [3].

The decreased risk in hospital mortality at high BMI levels is more controversial than the relationship between low BMI and mortality. Several studies showing an increased risk of death in patients with high BMI did not include general ICU patients [20], [22], [23], [32], [33], and the decreased risk of mortality at high BMI in critically patients has been confirmed in recent reports [34], [35], [36]. In Korean patients with ST segment elevation myocardial infarction undergoing primary percutaneous intervention, mortality was lowest in obese patients, although this association could be explained by the better use of guideline-recommended medical treatment, hemodynamic stability, and younger age in obese compared to non-obese patients. In the subgroup of patients with cardiovascular disease in our study, we also observed the lowest mortality among obese patients, but we did not have information on guideline-recommended medical treatment or hemodynamic stability. Finally, some of the studies that showed an increased risk of death in patients with high BMI in intensive care patients did not include general ICU patients [32], [33], [37], [38]. In our study, the lowest mortality was observed in patients in the highest BMI category in all patient subgroups except in surgical patients.

There is also increasing experimental evidence that adipocyte-secreted hormones, such as interleukin-10 [39], [40] and leptin [41], [42], [43], exert immunomodulatory effects that might decrease the inflammatory response and improve host survival. Physiologically, it is plausible that an excess of adipose tissue during a highly catabolic state, such as critical illness, may help prevent complications associated with critical illness.

This study has several limitations. “First, because this study used data from the VSKI study cohort [16], we did not have data on smoking history, an important determinant of BMI and mortality. We notice, however, that the proportion of smokers among Korean women is very low, and smoking is unlikely to have a major effect in this subgroup.” Second, the impact of pre-ICU fluid resuscitation on weight at ICU admission could not be quantified. Thus, it should be considered that the effect of processes of provided care such as fluid therapy might affect the association between BMI and hospital mortality [44]. Third, we could not assess the exact causal relationship between low BMI and mortality in this cohort. Thus, although comorbidities were adjusted for in the analyses, we cannot exclude the possibility that low BMI is a marker of poor health rather than a causal risk factor for increased mortality. Forth, few patients had BMI≥30.0, and the estimates in this group had wide confidence intervals. Finally, the study was performed in Korean ICUs; thus, the results may not be generalizable to other countries.

However, this study also has several strengths. First, we used a prospective design in a multicenter setting with wide representation of Korean ICUs. Second, the finding may have clinical implications, as BMI may add to the prognostic information in critically ill Korean patients and BMI may be considered to be an additional factor in risk prediction tools in ICU patients.

In conclusion, a graded inverse association between BMI and hospital mortality with a strong significant trend was found in critically ill patients admitted to a wide sample of ICUs in Korea. Additional research should be conducted to evaluate the mechanisms underlying this association and to determine whether BMI may contribute to the improved prediction of mortality for use in prognostic tools in critically ill patients.

## Supporting Information

Figure S1 **Multivariable-adjusted Cox-proportional hazard ratios with exact partial likelihood for hospital mortality comparing for body mass index categories.** Multivariable-adjusted Cox-proportional hazard ratios with exact partial likelihood and 95% confidence intervals are shown in this figure, illustrating a strong, graded, inverse association between BMI and hospital mortality, with a highly significant test for trend (*p*<0.001).
(TIF)

Table S1 **Baseline characteristics according to body mass index.**
(DOCX)

Table S2 **Severity and outcome according to body mass index.**
(DOC)

Table S3 **Cox-proportional hazard ratios with exact partial likelihood and 95% confidence intervals for hospital mortality according to body mass index categories.**
(DOC)
