# Supplementary figures and images for: Body Mass Index and Mortality in Korean Intensive Care Units: A Prospective Multicenter Cohort Study
Source: PLoS One. 2014 Apr 18;9(4):e90039. doi: 10.1371/journal.pone.0090039 (PMC3991578; doi:10.1371/journal.pone.0090039)

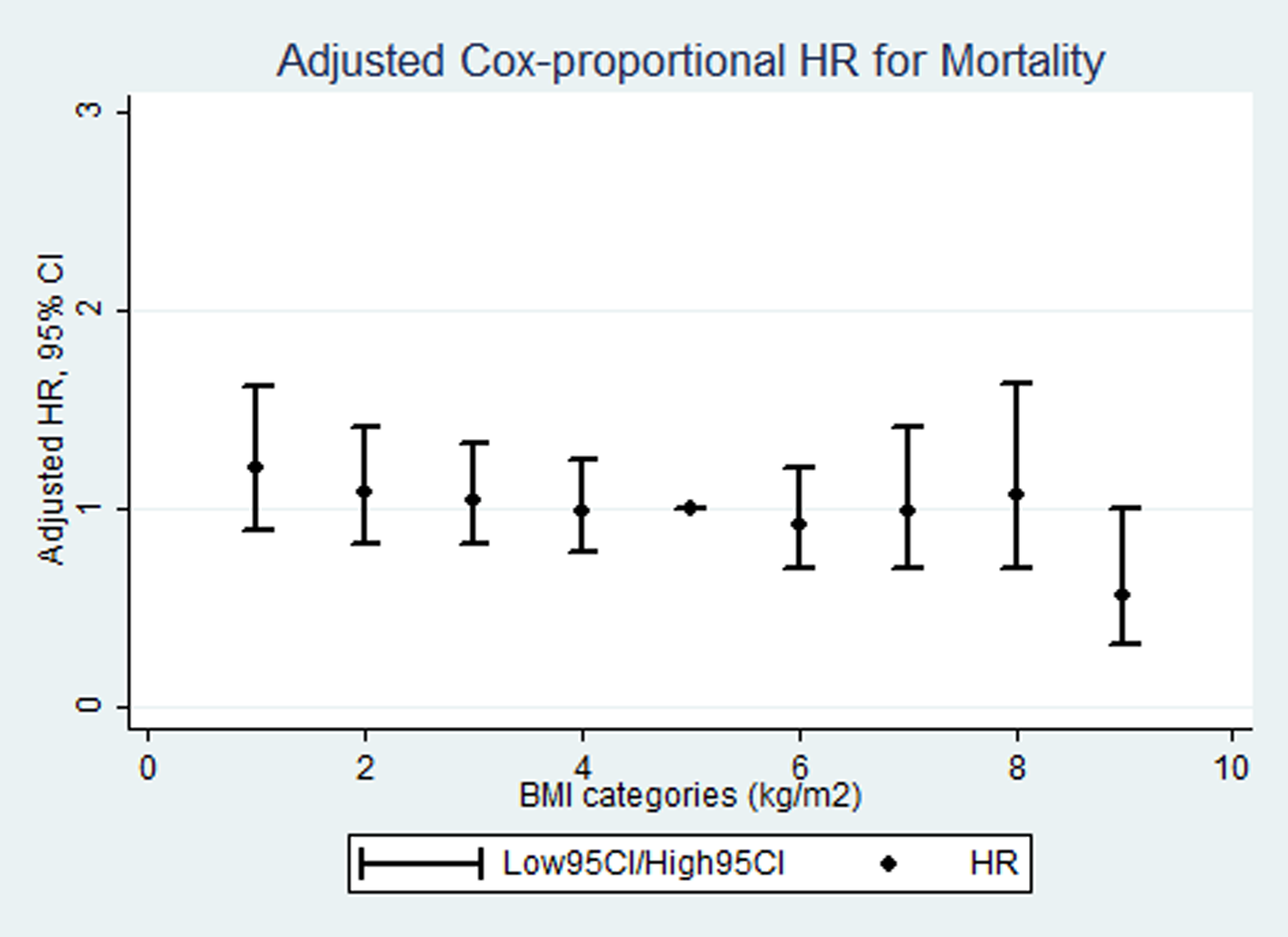

Supplement: Figure S1 — Multivariable-adjusted Cox-proportional hazard ratios with exact partial likelihood for hospital mortality comparing for body mass index categories. Multivariable-adjusted Cox-proportional hazard ratios with exact partial likelihood and 95% confidence intervals are shown in this figure, illustrating a strong, graded, inverse association between BMI and hospital mortality, with a highly significant test for trend (p<0.001). (TIF) [file pone.0090039.s001.tif]
