# Supplementary material for: Body Mass Index and Mortality in Korean Intensive Care Units: A Prospective Multicenter Cohort Study
Source: PLoS One. 2014 Apr 18;9(4):e90039. doi: 10.1371/journal.pone.0090039 (PMC3991578; doi:10.1371/journal.pone.0090039)
Supplement: Table S1 — Baseline characteristics according to body mass index. (DOCX) [file pone.0090039.s002.docx]

**Table S1 Baseline characteristics according to body mass index**

|  | **<17.0** | **17.0-18.9** | **19.0-20.9** | **21.0-22.9** | **23.0-24.9** | **25.0-26.9** | **27.0-28.9** | **29.0-30.9** | **≥31.0** | **Total** |
| --- | --- | --- | --- | --- | --- | --- | --- | --- | --- | --- |
| **Number, %** | 6.1 | 9.1 | 17 | 22.2 | 19.9 | 13.5 | 7.1 | 3.1 | 2 | 100 |
| **Median age** | 68 | 63 | 62 | 60 | 62 | 63 | 56 | 59 | 59 | 62 |
| **Male, %** | 62.4 | 61.6 | 65.1 | 64 | 66.7 | 64.4 | 62.9 | 59.3 | 41.9 | 63.8 |
| **Comorbidities, %** | | | | | | | | | | |
| Cirrhosis | 7.2 | 7.2 | 8.7 | 10 | 9.2 | 11.5 | 12.4 | 15.9 | 17.6 | 9.9 |
| Cardiovascular disease | 35.8 | 35.4 | 37.6 | 37.5 | 42.9 | 48.1 | 47.5 | 45.1 | 51.4 | 41 |
| CPF | 10.4 | 4.5 | 2.1 | 1.5 | 1.8 | 1.2 | 1.2 | 2.7 | 0 | 2.4 |
| DM | 16.7 | 2.01 | 18.3 | 24.5 | 20.5 | 26.1 | 18.9 | 27.4 | 29.7 | 21.8 |
| CRF | 10 | 9 | 9.3 | 9.1 | 7.7 | 6.9 | 7 | 7.1 | 8.1 | 8.4 |
| Cancer | 33.9 | 30.9 | 36.8 | 35.6 | 37.3 | 39.6 | 40.2 | 33.6 | 29.7 | 36.3 |
| **Status at ICU admission, %** | | | | | | | | | | |
| SS or septic shock | 22.6 | 21.6 | 16.9 | 15.7 | 15.3 | 16 | 11.6 | 14.2 | 27 | 16.7 |
| ALI or ARDS | 11.3 | 9. | 7.2 | 6.8 | 7.6 | 4.9 | 4.3 | 3.5 | 6.8 | 7 |
| **Admission category, %** | | | | | | | | | | |
| Medical | 77.8 | 65.8 | 60.8 | 60.8 | 53.1 | 53.7 | 51.4 | 55.8 | 62.2 | 59 |
| Surgical | 22.2 | 34.2 | 39.2 | 39.2 | 46.9 | 46.3 | 48.7 | 44.3 | 37.8 | 41 |
| **Reason for ICU admission, %** | | | | | | | | | | |
| Observational | 28.5 | 39.3 | 42.4 | 44 | 44.8 | 46.7 | 47.1 | 34.5 | 33.8 | 42.6 |
| Cardiovascular | 18.1 | 14.4 | 11.3 | 12.8 | 12.4 | 11.9 | 13.9 | 16.8 | 21.6 | 13.2 |
| Digestive | 3.6 | 5.4 | 5 | 6 | 4.3 | 5.7 | 5 | 6.2 | 5.4 | 5.2 |
| Hepatic failure | 1.8 | 2.4 | 4.3 | 2.7 | 4.5 | 5.1 | 7.7 | 8 | 9.5 | 4.2 |
| Neurologic | 2.7 | 4.2 | 7.1 | 5.7 | 5.8 | 6.1 | 6.2 | 4.4 | 4.1 | 5.6 |
| Renal | 1.8 | 1.5 | 2.7 | 1.6 | 1.9 | 1.6 | 0.4 | 2.7 | 4.1 | 1.9 |
| Respiratory | 38.5 | 26.4 | 19.1 | 16.2 | 17.2 | 13.1 | 11.6 | 13.3 | 14.9 | 18.3 |

CPF, chronic pulmonary disease; DM, diabetes mellitus; CRF, chronic renal failure; ICU, intensive care unit; SS, severe sepsis; ALI, acute lung injury; ARDS, acute respiratory distress syndrome.
