# Supplementary material for: Body Mass Index and Mortality in Korean Intensive Care Units: A Prospective Multicenter Cohort Study
Source: PLoS One. 2014 Apr 18;9(4):e90039. doi: 10.1371/journal.pone.0090039 (PMC3991578; doi:10.1371/journal.pone.0090039)
Supplement: Table S2 — Severity and outcome according to body mass index. (DOC) [file pone.0090039.s003.doc]

**Table S2 Severity and outcome according to body mass index (N = 3655**)

|  | **<17.0** | **17.0-18.9** | **19.0-20.9** | **21.0-22.9** | **23.0-24.9** | **25.0-26.9** | **27.0-28.9** | **29.0-30.9** | **≥31.0** | **Total** |
| --- | --- | --- | --- | --- | --- | --- | --- | --- | --- | --- |
| **SAPS 3** | 60 | 57 | 54 | 53 | 52 | 53 | 51 | 51 | 58.5 | 54 |
| **PDR** | 35.6 | 29.5 | 23.9 | 22.1 | 20.5 | 22.1 | 18.9 | 18.9 | 32.5 | 23.9 |
| **SOFA** | 7 | 7 | 6 | 6 | 6 | 5 | 6 | 6 | 8.5 | 6 |
| **ICU LOS** | 4 | 5 | 4 | 4 | 4 | 3 | 4 | 5 | 4 | 4 |
| **Hospital LOS** | 16 | 22 | 19 | 18 | 18 | 17 | 18 | 17 | 18 | 18 |
| **ICU mortality** | 22.2 | 20.4 | 15.8 | 14.4 | 12.8 | 13.3 | 13.5 | 13.3 | 13.5 | 15.1 |
| **Hospital mortality** | 33.5 | 28.5 | 22 | 21.2 | 18.2 | 19 | 16.6 | 23 | 17.6 | 21.5 |

Continuous variables are presented as median.

Categorical variables are presented as %.

SAPS, simplified acute physiology score; PDR, predicted death rate; SOFA, sequential organ failure assessment; ICU, intensive care unit; LOS, length of stay.
