# Supplementary material for: Body Mass Index and Mortality in Korean Intensive Care Units: A Prospective Multicenter Cohort Study
Source: PLoS One. 2014 Apr 18;9(4):e90039. doi: 10.1371/journal.pone.0090039 (PMC3991578; doi:10.1371/journal.pone.0090039)
Supplement: Table S3 — Cox-proportional hazard ratios with exact partial likelihood and 95% confidence intervals for hospital mortality according to body mass index categories. (DOC) [file pone.0090039.s004.doc]

**Table S3 Cox-proportional hazard ratios with exact partial likelihood and 95% confidence intervals for hospital mortality according to body mass index categories**

|  | **<17.0** | **17.0-18.9** | **19.0-20.9** | **21.0-22.9** | **23.0-24.9** | **25.0-26.9** | **27.0-28.9** | **29.0-30.9** | **≥31.0** |
| --- | --- | --- | --- | --- | --- | --- | --- | --- | --- |
| **Total (N = 3655)** | | | | | | | | | |
| Number of deaths, % | 33.5 | 28.5 | 22 | 21.2 | 18.2 | 19 | 16.6 | 23 | 17.6 |
| Unadjusted HR | 1.611.21 | 1.31 | 1.1 | 1.13 | 1 | 1.06 | 1 | 1.1 | 0.9 |
| (95% CI) | (1.21-2.16) | (1.01-1.71) | (0.87-1.4) | (0.9-1.42) | (reference) | (0.82-1.39) | (0.7-1.41) | (0.72-1.68) | (0.51-1.59) |
| Adjusted HR^*^ | 1.21 | 1.09 | 1.04 | 0.99 | 1 | 0.92 | 0.99 | 1.07 | 0.57 |
| (95% CI) | (0.9-1.62) | (0.83-1.42) | (0.82-1.33) | (0.79-1.25) | (reference) | (0.7-1.21) | (0.7-1.41) | (0.7-1.64) | (0.32-1.01) |
| **Patients with cardiovascular disease (N = 1497)** | | | | | | | | | |
| Number of deaths, % | 29.1 | 31.4 | 26.5 | 20.7 | 20.8 | 20.2 | 15.5 | 21.6 | 13.2 |
| Unadjusted HR | 1.26 | 1.12 | 1.04 | 0.86 | 1 | 1.05 | 0.76 | 1.13 | 0.59 |
| (95% CI) | (0.78-2.04) | (0.75-1.68) | (0.73-1.47) | (0.61-1.22) | (reference) | (0.72-1.53) | (0.45-1.27) | (0.6-2.15) | (0.23-1.46) |
| Adjusted HR† | 1.14 | 0.92 | 1.01 | 0.85 | 1 | 1.01 | 0.9 | 0.93 | 0.38 |
| (95% CI) | (0.7-1.86) | (0.6-1.39) | (0.71-1.45) | (0.6-1.22) | (reference) | (0.69-1.49) | (0.53-1.52) | (0.48-1.79) | (0.15-0.96) |
| **Patients with acute respiratory failure (N = 669)** | | | | | | | | | |
| Number of deaths, % | 47.1 | 38.6 | 38.7 | 45 | 41.6 | 35.4 | 33.3 | 33.3 | 27.3 |
| Unadjusted HR | 1.13 | 0.85 | 0.94 | 1.12 | 1 | 0.89 | 1.05 | 0.92 | 0.72 |
| (95% CI) | (0.74-1.72) | (0.55-1.31) | (0.63-1.4) | (0.77-1.63) | (reference) | (0.54-1.46) | (0.53-2.07) | (0.36-2.31) | (0.22-2.32) |
| Adjusted HR§ | 1.08 | 0.81 | 0.99 | 1 | 1 | 0.7 | 0.83 | 0.85 | 0.6 |
| (95% CI) | (0.7-1.66) | (0.52-1.26) | (0.66-1.49) | (0.68-1.48) | (reference) | (0.42-1.17) | (0.41-1.66) | (0.33-2.18) | (0.18-1.99) |
| **Surgical patients (N = 1499)** | | | | | | | | | |
| Number of deaths, % | 10.2 | 13.2 | 6.6 | 7.2 | 5 | 7.4 | 5.6 | 12 | 7.1 |
| Unadjusted HR | 1.69 | 1.9 | 1.13 | 1.37 | 1 | 1.71 | 1.18 | 1.56 | 0.97 |
| (95% CI) | (0.61-4.69) | (0.94-3.81) | (0.57-2.24) | (0.73-2.57) | (reference) | (0.87-3.36) | (0.49-2.85) | (0.6-4.04) | (0.22-4.2) |
| Adjusted HR‡ | 1.93 | 1.87 | 1.3 | 1.24 | 1 | 1.61 | 0.97 | 1.49 | 1.33 |
| (95% CI) | (0.65-5.69) | (0.93-3.79) | (0.65-2.58) | (0.66-2.36) | (reference) | (0.82-3.2) | (0.4-2.36) | (0.56-3.96) | (0.3-5.89) |
| **Medical patients (N = 2156)** | | | | | | | | | |
| Number of deaths, % | 40.1 | 36.5 | 32 | 30.2 | 29.8 | 29 | 27.1 | 31.8 | 23.9 |
| Unadjusted HR | 1.21 | 1.07 | 0.99 | 0.99 | 1 | 0.94 | 1.08 | 1.14 | 0.86 |
| (95% CI) | (0.89-1.63) | (0.8-1.42) | (0.76-1.28) | (0.78-1.27) | (reference) | (0.71-1.26) | (0.74-1.57) | (0.71-1.84) | (0.46-1.6) |
| Adjusted HR# | 1.13 | 1 | 1.01 | 0.93 | 1 | 0.83 | 1.02 | 0.91 | 0.51 |
| (95% CI) | (0.83-1.53) | (0.75-1.34) | (0.78-1.32) | (0.73-1.19) | (reference) | (0.61-1.11) | (0.7-1.5) | (0.56-2.48) | (0.27-0.97) |

HR, hazard ratio; CI, confidence interval

^*^Adjusted for age, sex, SOFA, CPF, DM, cancer, severe sepsis or septic shock at ICU admission, ARDS at ICU admission, admission category, use of CRRT, mechanical ventilation, and use of vasopressors.

†Adjusted age, sex, SOFA, DM, cancer, severe sepsis or septic shock at ICU admission, ARDS at ICU admission, admission category, use of CRRT, mechanical ventilation, and use of vasopressors.

§Adjusted age, sex, SOFA, cirrhosis, CPF, cancer, severe sepsis or septic shock at ICU admission, ARDS at ICU admission, admission category, use of CRRT, mechanical ventilation, and use of vasopressors.

‡Adjusted age, sex, SOFA, cirrhosis, cancer, severe sepsis or septic shock at ICU admission, ARDS at ICU admission, use of CRRT, mechanical ventilation, and use of vasopressors.

#Adjusted age, sex, SOFA, cirrhosis, CPF, cancer, severe sepsis or septic shock at ICU admission, ARDS at ICU admission, use of CRRT, mechanical ventilation, and use of vasopressors.
